# Stability of diagnostic rate in a cohort of 38,813 colorectal polyp specimens and implications for histomorphology and statistical process control

**DOI:** 10.1038/s41598-021-95862-2

**Published:** 2021-08-20

**Authors:** Michael Bonert, Asghar Naqvi, Mozibur Rahman, John K. Marshall, Ted Xenodemetropoulos, Paul Arora, Justin Slater, Pierre Major

**Affiliations:** 1 Department of Pathology and Molecular Medicine, McMaster University, Hamilton, Canada; 2 Department of Pathology, St. Joseph’s Healthcare Hamilton, Room L206, 50 Charlton Avenue East, Hamilton, ON L8N 4A6 Canada; 3 Westchester Medical Center, Valhalla, USA; 4 Department of Medicine, Farncombe Family Digestive Health Research Institute, McMaster University, Hamilton, Canada; 5 Division of Gastroenterology, Department of Medicine, McMaster University, Hamilton, Canada; 6 Division of Epidemiology, Dalla Lana School of Public Health, University of Toronto, Toronto, Canada; 7 Lighthouse Outcomes Inc., Toronto, Canada; 8 Juravinski Hospital/Hamilton Health Sciences Centre, Hamilton, Canada; 9 Department of Oncology, McMaster University, Hamilton, Canada

**Keywords:** Pathology, Statistics, Biomedical engineering, Quality control

## Abstract

This work sought to quantify pathologists’ diagnostic bias over time in their evaluation of colorectal polyps to assess how this may impact the utility of statistical process control (SPC). All colorectal polyp specimens(CRPS) for 2011–2017 in a region were categorized using a validated free text string matching algorithm. Pathologist diagnostic rates (PDRs) for high grade dysplasia (HGD), tubular adenoma (TA_ad), villous morphology (TVA + VA), sessile serrated adenoma (SSA) and hyperplastic polyp (HP), were assessed (1) for each pathologist in yearly intervals with control charts (CCs), and (2) with a generalized linear model (GLM). The study included 64,115 CRPS. Fifteen pathologists each interpreted > 150 CRPS/year in all years and together diagnosed 38,813. The number of pathologists (of 15) with zero or one (*p* < 0.05) outlier in seven years, compared to their overall PDR, was 13, 9, 9, 5 and 9 for HGD, TVA + VA, TA_ad, HP and SSA respectively. The GLM confirmed, for the subset where pathologists/endoscopists saw > 600 CRPS each(total 52,760 CRPS), that pathologist, endoscopist, anatomical location and year were all strongly correlated (all *p* < 0.0001) with the diagnosis. The moderate PDR stability over time supports the hypothesis that diagnostic rates are amendable to calibration via SPC and outcome data.

## Introduction

Diagnostic variance can be measured in several ways. In pathology, it is traditionally measured with kappa values generated by small data sets interpreted by a variable number of pathologists. Inter-rater agreement is inherently dependent on both the tissue type and the clinical diagnosis, with the usual results being poor to moderate. The gold standard diagnosis is commonly determined through the consensus of a panel of experts, rather than hard outcomes-driven data^1^.

From the perspective of manufacturing industries (where defect rates are commonly measured in parts per thousand or parts per million), significant disagreements/errors in pathology (that change the management) are common^2^. Such high error rates are often rationalized by the inherently imprecise nature of histomorphology and the innate difficulty in achieving high levels of precision within a complex system such as the human body. The later is a historical and longstanding philosophical debate: anti-reductionism (the body is impossible to subdivide effectively into components that enhance understanding) versus reductionism (the body can be subdivided effectively into components that enhance understanding).

The precision/lack of precision in histomorphology is the topic of this study. In the context of the above-mentioned philosophical debate, we support a reductionist approach of subdivision into component elements can allow for enhanced understanding with sufficient determination, and appropriate models, statistical methods and process management. As such, if diagnostic variation is explained by consistent bias among healthcare providers (as opposed to large swings in the diagnostic rates/diagnostic instability), it should be amendable to an intervention that (deconstructs the diagnostic process), reduces variation and, with calibration (including pathologist rate awareness), may be used to more appropriately stratify patients and improve outcomes.

We, thus, hypothesize that histomorphology is actually much more reproducible than seen in most practise environments, and that a lack of “control” within a practice environment (rather than imprecision in histomorphology) is the basis of the considerable diagnostic variation in pathological reporting encountered. The specific goals of this study are to (1) understand diagnostic variance among pathologists and (2) compare diagnostic variance using a population-based approach.

Population-based comparisons are predicated on the assumptions of (1) population disease stability (i.e. population disease characteristics are stable over time) and (2) the absence of a selection/case assignment bias for the interpreting pathologist. The first assumption can be determined to some degree from the data itself, if the sample is sufficiently large for the time frame. The second assumption depends on the practice environment/case distribution. Prior work in our laboratory found very similar call rates for *Helicobacter pylori* gastritis^3^ and endoscopic bronchial ultrasound-fine needle aspirations^4^. The simplest and most likely explanation is that the case assignment in our practice environment is effectively random.

Statistical process control (SPC) was first applied in medicine by pathologists in the 1960s^5^, and there is now a substantial literature on SPC in medicine^6^ and it is used in clinical pathology. Although the SPC methodology has not routinely been applied in anatomical pathology, it should be an effective tool if there is approximate diagnostic stability and approximate disease stability over time^7^. “Appendix E” briefly explains SPC and why demonstrating disease stability and diagnostic rate stability (in the context of significant diagnostic rate differences) would be sufficient to suggest SPC should be effective.

Colorectal polyp specimens were chosen for study as (1) they are a high-volume specimen familiar to pathology generalists and subspecialists, and (2) interpretative differences are usually of low or moderate consequence; the questions are frequently one benign diagnosis versus another benign diagnosis (rather than benign versus malignant).

## Methods

Research ethics board approval was obtained (Hamilton Integrated Regional Ethics Board (HiREB)—HiREB# 2016-2295-C and HiREB# 2018-4445-C) with sign off from the laboratory director. The study was done in accordance with national ethics guidelines and relevant regulations. This study had no research subjects; thus, the requirement for informed consent from subjects is not applicable. All in house surgical pathology reports accessioned from January 2011 to December 2017 were extracted from the Laboratory Information System.

Extracted reports were stripped of all patient identifiers. Custom computer programs written in the Python programming language then reconstructed the report structure, subdivided cases into parts, and allowed complex searches within cases and parts.

Colorectal polyps specimens which met the following criteria were retrieved:

1. one of the following words: “colon”, “rectum”, “rectal”, “cecum”, “cecal”, “rectosigmoid” in the “source of specimen” section of the report
2. “polyp” within the “source of specimen” section of the report

The “source of specimen” section is what the endoscopist labels the specimens as. It was chosen as it was deemed to have the most uniformity of the report sections.

It should be noted the “specimens” correspond to bottles/containers submitted. One specimen may in fact contain zero, one or several polyps that are from one or more anatomical sites. Several specimens may be derived from one surgical case; quantification of this is not part of the study. A number of surgical cases may originate from one individual; however, quantification of this is not part of the study.

Retrieved specimens were written to a tab separated file which was then further processed to replace the surgical case number, submitting physicians and pathologists with unique anonymous identifiers.

Specimens were then tabulated.

Cases were classified by fuzzy string matching using an open source library called *google-diff-patch-match* and several dictionaries of terms into:

1. one or more 40 diagnostic categories (based on 194 phrases) – see “Appendix A”
2. one or more 12 anatomical locations (based on 18 phrases or 9 measurement cut points) – see “Appendix B”

Audits were done with randomly selected CRPS to assess the accuracy of the computer’s classification. This involved pathologists comparing the (pathology) report free text with the diagnostic categories (listed in “Appendix A” and “Appendix B”) assigned by the computer.

After categorization and tabulation, the anonymized data set was further processed by a custom GNU/Octave^8^ program to create funnel plots and control charts. Funnel plots that included data from all pathologists were centred on the group median diagnostic rate (GMDR). The GMDR was chosen, as the reference, as it is (1) not influenced by significant outliers, and (2) not biased by case volume. The funnel edges were defined by two and three standard deviations from the GMDR and calculated via the normal approximation of the binomial distribution as previously described^9^. Control charts (equivalent to the funnel plots) were created by normalizing to the number of cases read by the highest volume pathologist in the group; details of the normalization are within “Appendix C” ^15^. Normalization was done to obscure case volume and facilitate ease of interpretation.

Pathologist-specific control charts (showing the year-to-year variation) were created with the individual pathologist’s mean diagnostic rate, if the pathologist interpreted at least 600 specimens. The mean was chosen as the number of cases per year was not equal; using the mean ensured that the cases had equal weight in determining the control chart “centre”. Data points for a given year were plotted only if the pathologist interpreted at least 150 specimens in that year. The thresholds (600 specimens, 150 specimens/year) were chosen to ensure that the PDR estimates are have relatively narrow confidence intervals.

Generalized linear models, with a random intercept for each hospital, were utilized to estimate the association between independent variables (pathologist, submitting MD, anatomical location, and year) and high-grade dysplasia (HGD), villous component (TVA + VA), hyperplastic polyp (HP), tubular adenoma (TA), and sessile serrated adenoma (SSA). These models were implemented using SAS version 9.4 (SAS Institute, Cary, NC).

Prior to this calculation, all pathologists and all submitting physicians interpreting or submitting less than 600 specimens were excluded from the dataset. Uncommon nonspecific/vague anatomical sites (e.g. “anastomosis [not otherwise specified]” or “left colon [not otherwise specified]”) were also excluded from the data set to avoid the possibility of over-fitting.

### Ethics approval and consent to participate

Ethics approval was obtained. Consent for publication is not applicable.

## Results

In the study period, the program extracted 64,115 colorectal polyp specimens. A small number of polyp specimens (< 1%) may not have been captured, as we previously did an analysis on this (published in abstract form^9^ in a cohort of 11,457 large bowel polyp specimens, 68 surgical cases could not be parsed (separated into parts/specimens). The 68 cases (not parsed by the computer) were examined in detail and it was determined that 37 had unusual report formatting (e.g. parts were out of order), 24 had a mislabelled part (e.g. “Part D” transcribed as “Part P”), 7 had missing specimen parts (e.g. requisition has Parts A-C, diagnosis sections has Part A-B (Part C is absent)).

In the 64,115 colorectal polyp specimens that were retrieved, the hierarchical free text string matching algorithm (HFTSMA) could classify 63,050 of the specimens with regard to a diagnosis, and 63,508 with regard to the anatomical site. Several individuals independently assessed the accuracy of the computer’s classification via random audits in at least 789 specimens. Prior audits suggested that the overall accuracy is ~ 97%.

The three percent that is not classified correctly is mostly not classifiable; we previously analyzed 55 of 92 unclassified colorectal polyp specimens in a cohort of 11,457 large bowel polyp specimens^9^. In most cases, the failure was nontechnical/unrelated to the HFTSMA; 19 cases were rare/descriptive diagnoses, 24 vaguely worded diagnoses, 7 failed due to (unusual) report formatting/transcription and 5 failed for an unknown reason.

Since the custom analysis programs have evolved in the past 2 years, we did a further random audit of the computer’s classification. Four hundred polyp specimens were selected at random and the computer-generated diagnostic codes were compared to the text of the diagnosis. In this analysis, 394 cases were correctly classified and 6 not coded; this matched our prior experience. We also recently examined sessile serrated adenomas (SSA) over multiple years in a subset of ~ 7000 colorectal polyp specimens^10^. In that context, the accuracy of SSA classification was examined; in 400 randomly selected cases there were zero errors in the classification of SSA/not SSA. Report auditing (based on the results) found systematic misclassifications in HGD and TA; these were corrected by adjusting the dictionary of diagnostic terms and re-running the analysis.

Outliers > 7 SDs from the GMDR (seen in SSA, HP and TVA + VA) prompted reviews of 100–200 randomly selected specimen reports for each of the anonymous outlier pathologists, and these confirmed that there is no significant categorization error (due to unusual reporting language) from the HFTSMA that could explain the observed diagnostic rates.

An overview of the colorectal polyp cohort is found within Table S1 (see supplemental materials). ‘Adenoma [not otherwise specified]’ was combined with ‘tubular adenoma’, as these appeared to be used as synonyms by a subset of pathologists.

**Figure 1:**
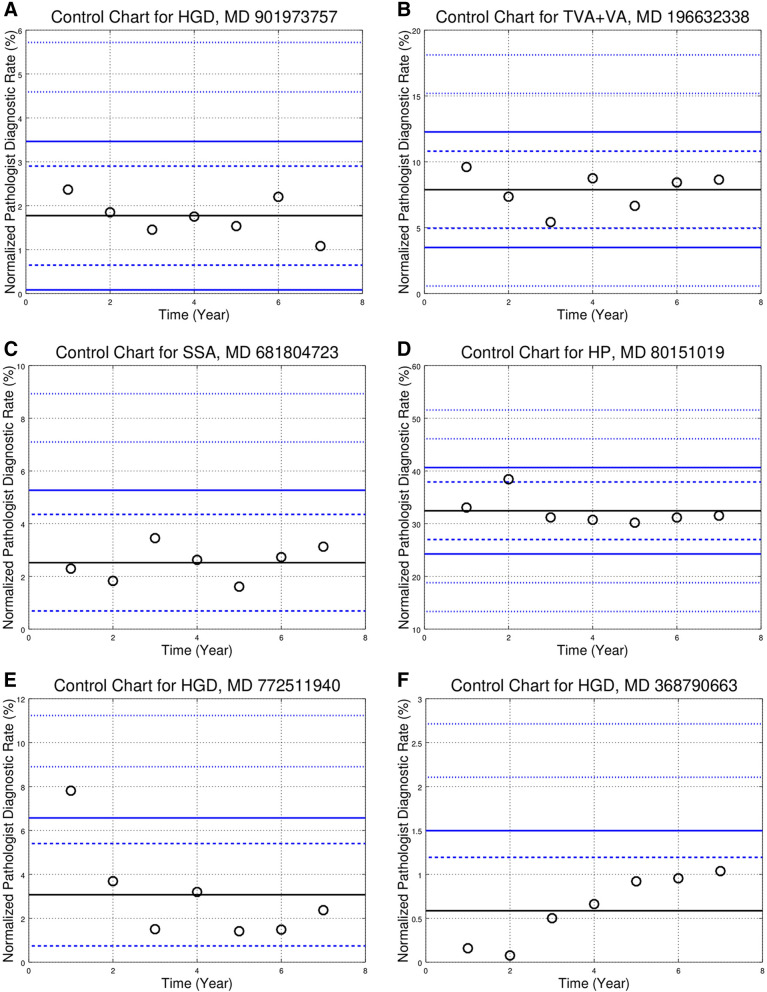

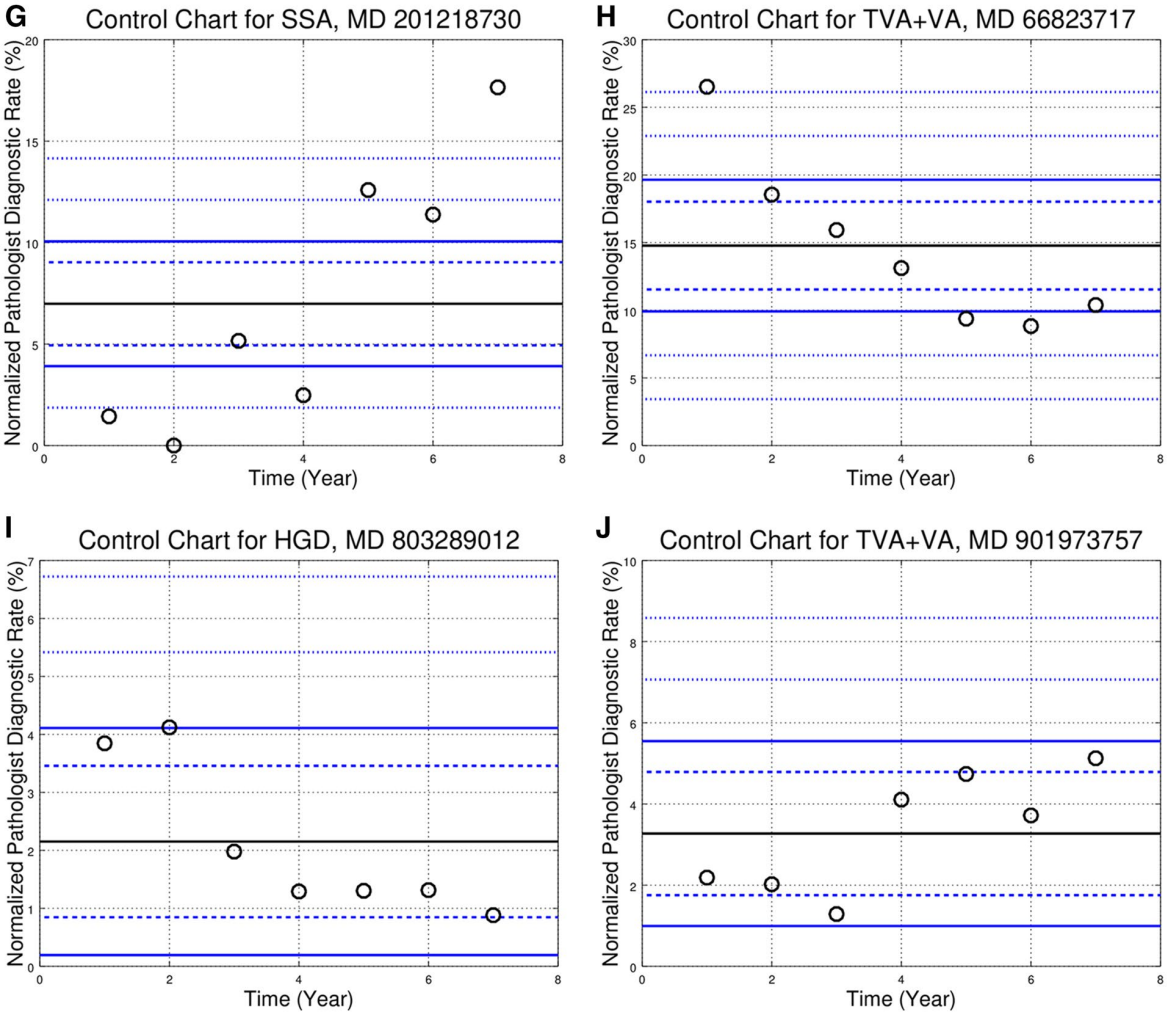
Control charts for individual pathologists. Each panel (control chart) in this figure is one diagnosis (e.g. SSA) for a pathologist that read > 150 CRPS/year in the seven years of the study. The black circles represent the normalized PDR in different years; the denominator within the normalized PDR is the number of colorectal polyp specimens read by the individual pathologist in the year. The thick solid black line is the pathologist’s average (or mean) call rate (PACR). The dashed blue control lines above and below the PACR are 2 standard deviations (SDs) from the PACR; points outside the range are *p* < 0.05. The solid blue control lines represent 3 SDs from the PACR; points outside the range are *p* < 0.003. The outer finely dashed blue control lines above/below the PACR are 5 SDs and 7 SDs from the PACR respectively; points above/below the lines are *p* < 6e−7 and *p* < 3e−12 respectively. Controls lines may be absent if the PACR is close to zero (as negative normalized PDRs do not have a physical interpretation). The individual panels (control charts) are (**A**) high-grade dysplasia (HGD) showing the “in control” condition; (**B**) tubulovillous adenoma + villous adenoma (TVA + VA) showing the “in control” condition; (**C**) sessile serrated adenoma (SSA) showing the “in control” condition; (**D**) hyperplastic polyp (HP) with a “blip” (> 2 SDs/*P* < 0.05 outlier) in year two of the study; (**E**) HGD with a “blip” (*P* < 0.003 outlier) in year one of the study; (**F**) HGD with a “trend” (non-significant, not crossing control lines); (**G**) SSA with a “trend” (significant, crossing control lines); (**H**) TVA + VA with a “trend” (significant, crossing control lines); (**I**) HGD with a “step” (significant, crossing control lines); (**j**) TVA + VA with a “step” (significant, crossing control lines).

The control chart showed various patterns. The “in control” pattern was common, and is the expected result if (1) the individual pathologist has not changed their practice, (2) the population disease rates are stable. Representative control charts of this type are seen in Fig. 1A–C.

Some control charts (e.g. Figure 1D,E) showed an outlier in the background of what would otherwise be “in control”; this was the most common pattern (See Table 2). A third type of chart shows a pattern (increasing or decreasing) with or without crossing control lines (e.g. Fig. 1F–H). A fourth type of chart shows a step (upward or downward) with relative stability before and afterward (e.g. Fig. 1I,J).

The control charts constructed around the pathologist’s mean PDR are summarized in Table 1a.

**Table 1:** (Subsection **a**) Comparison of pathologists to self. Number of outlier years for all pathologists with seven years of data. There are 15 pathologists with 7 years of data; thus, there are 105 pathologist x years, (Subsection **b**) Comparison to pathologists to self (normalized). Fraction that are outliers for control charts centred on the pathologist’s mean diagnostic rate. The numbers in this table are generated by dividing through by the total number of pathologist x years (105), e.g. 9/105 = 0.09 for HGD 2 SD. SD = standard deviation.

Variation	HGD	TVA + VA	TA_ad	HP	SSA
Subsection **a**					
< 2 SD	96	81	82	72	71
> 2 SD	9	24	23	33	34
> 3 SD	2	10	8	10	18
> 5 SD	0	2	1	0	10
> 7 SD	0	1	0	0	5
PY (Total)	105	105	105	105	105

Variation	HGD	TVA + VA	TA_ad	HP	SSA
Subsection **b**					
< 2 SD	0.91	0.77	0.78	0.69	0.68
> 2 SD	0.09	0.23	0.22	0.31	0.32
> 3 SD	0.02	0.10	0.08	0.10	0.17
> 5 SD	0.00	0.02	0.01	0.00	0.10
> 7 SD	0.00	0.01	0.00	0.00	0.05

*HGD* high-grade dysplasia, *HP* hyperplastic polyp, *SSA* sessile serrated adenoma, *TVA + VA* tubulovillous adenoma + villous adenoma, *TA_ad* tubular adenoma + adenoma NOS, *PY* pathologist-years; 15 pathologists × 7 years = 105 pathologist × years.

**Figure 2:**
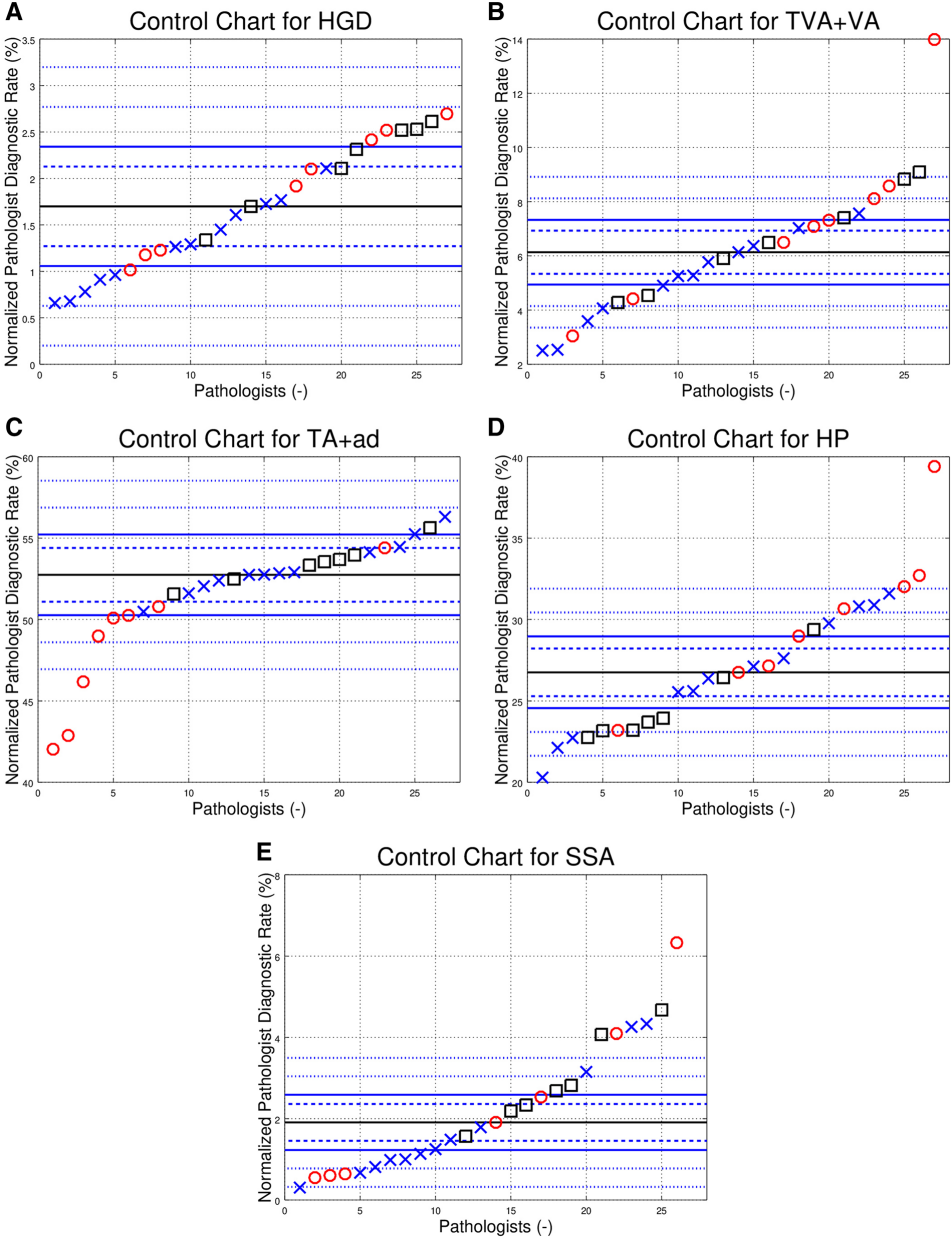
Control charts showing the normalized pathologist diagnostic rates (PDRs) for the 27 pathologists reading > 600 CRPS in the seven year study period. Each panel (control chart) in this figure is one diagnosis, e.g. SSA. The different markers (red circles, blue Xs, black boxes) represent individual pathologists from different hospitals. The solid black line is the group median diagnostic rate (GMDR). The dashed blue control lines above and below the GMDR are 2 standard deviations (SDs) from the GDMR; pathologists outside the inner funnel are statistically different than the GMDR (*p* < 0.05). The solid blue control lines above and below the GMDR represent 3 SDs; pathologist outside the outer funnel are statistically different than the GMDR (*p* < 0.003). The outer finely dashed blue control lines above/below the GMDR are 5 SDs and 7 SDs from the GMDR respectively; points above/below the lines are *p* < 6e−7 and *p* < 3e−12 respectively. Controls lines may be absent if the GMDR is close to zero (as negative normalized PDRs do not have a physical interpretation). The individual panels (control charts) are (**A**) normalized high-grade dysplasia (HGD) PDRs; (**B**) normalized tubulovillous adenoma + villous adenoma (TVA + VA) PDRs; (**C**) normalized tubular adenoma and adenoma not otherwise specified (TA_ad) PDRs; (**D**) normalized hyperplastic polyp (HP) PDRs; (**E**) normalized sessile serrated adenoma (SSA) PDRs, only the 26 of the 27 pathologists shown; one pathologist had a normed diagnostic rate > 25% (not shown).

The control charts, centred on the group median diagnostic rate (GMDR), showed many outliers (see Fig. 2A–E), and are summarized in Table 3a. Outliers (*p* < 0.05) were calculated using the GMDR for each of the hospitals. The results are in Table 3c; specimens from two hospitals are effectively shared by one group of pathologists; thus, this was considered one site for the purpose of the control chart analysis (Table 2).

**Table 2:** Comparison of pathologists to self. Number of pathologists by the number of (> 2 standard deviation) outliers in relation to each pathologist’s mean call rate over the seven-year period. This tabulation shows the number of 2 SD outliers they had for each diagnosis, e.g. 8 pathologists had zero outlier years for HGD, 1 pathologist had 5 outlier years for TVA + VA (this is shown in Fig. 1H), 2 pathologists had 4 outlier years for SSA, 2 pathologists had 6 outlier years for SSA (one of the two pathologists is shown in Fig. 1G).

# Outliers	HGD	TVA + VA	TA_ad	HP	SSA
0	8	6	3	0	2
1	5	3	6	5	7
2	2	3	2	5	1
3	0	1	3	2	0
4	0	0	1	3	2
5	0	1	0	0	1
6	0	0	0	0	2
7	0	1	0	0	0
Sum	15	15	15	15	15

*HGD* high-grade dysplasia, *HP* hyperplastic polyp, *SSA* sessile serrated adenoma, *TVA+VA* tubulovillous adenoma + villous adenoma, *TA_ad* tubular adenoma + adenoma NOS, *P* number of pathologists.

**Table 3:** Subsection **a** Comparison of pathologists to the 27 pathologists that read > 600 CRPS. Number of outlier pathologists based on the group median diagnostic rate. The number of “ < 2 SD” and “ > 2 SD” pathologists total 27. Subsection **b** Comparison of pathologists to the 27 pathologists that read > 600 CRPS (normalized). Fraction that are outliers for control charts around the group median diagnostic rate. The numbers in this table are generated by dividing through by the total number of pathologists (27). Subsection **c** Comparison of pathologists to the hospital site. Number of outlier pathologists based on the group median for the individual hospital sites. Subsection **d** Comparison of pathologists to the hospital site (normalized). Fraction that are outliers for control charts around the group median diagnostic rate for the each of the hospital sites. The numbers in this table are generated by dividing through by the total number of pathologists (27).

Variation	HGD	TVA + VA	TA_ad	HP	SSA
Subsection ** a**					
< 2 SD	11	6	14	8	6
> 2 SD	16	21	13	19	21
> 3 SD	12	16	9	19	19
> 5 SD	0	9	3	11	13
> 7 SD	0	5	3	4	8
P (Total)	27	27	27	27	27

Variation	HGD	TVA + VA	TA_ad	HP	SSA
Subsection **b**					
< 2 SD	0.41	0.22	0.52	0.30	0.22
> 2 SD	0.59	0.78	0.48	0.70	0.78
> 3 SD	0.44	0.59	0.33	0.70	0.70
> 5 SD	0.00	0.33	0.11	0.41	0.48
> 7 SD	0.00	0.19	0.11	0.15	0.30

Variation	HGD	TVA + VA	TA + adN	HP	SSA
Subsection **c**					
< 2 SD	16	9	17	11	11
> 2 SD	11	18	10	16	16
> 3 SD	6	14	5	14	15
> 5 SD	1	10	3	10	12
> 7 SD	1	3	2	4	7
P (Total)	27	27	27	27	27

Variation	HGD	TVA + VA	TA + adN	HP	SSA
Subsection **d**					
< 2 SD	0.59	0.33	0.63	0.41	0.41
> 2 SD	0.41	0.67	0.37	0.59	0.59
> 3 SD	0.22	0.52	0.19	0.52	0.56
> 5 SD	0.04	0.37	0.11	0.37	0.44
> 7 SD	0.04	0.11	0.07	0.15	0.26

*HGD* high-grade dysplasia, *HP* hyperplastic polyp, *SSA* sessile serrated adenoma, *TVA + VA* tubulovillous adenoma + villous adenoma, *TA_ad* tubular adenoma + adenoma NOS, *P* number of pathologists.

The control charts based on the individual pathologist’s mean PDR and those based on the GMDR are not directly comparable; however, the summary data (Tables 1a and 3a) does allow some comparison. The fraction of outliers (shown in Tables 1b, 3b,d) were calculated using the total number of elements—105 and 27 respectively. These tables show that there are proportionally less outliers when the data is plotted by the pathologist, suggesting the individual pathologist is a very strong predictor—a result demonstrated with logistic regression. For example, the fraction > 2 SD for HGD is 0.09, 0.41 and 0.59 for comparison to self (Table 1a), comparison to hospital site (Table 3b) and comparison to the group of 27 pathologists (Table 3d) respectively; this is also shown in Table S2 (see supplemental materials).

The outlier frequencies within Table 1a (with the exception of HGD) are highly improbable to be only a consequence of sampling. The cumulative probability of being outside two standard deviations for (1) the number of outliers and (2) all greater number of outliers for HGD is *p* ~ 0.08 (see “Appendix D” for details). The probability of being outside two standard deviations (for (1) the number of outliers and (2) all greater number of outliers) for all the other diagnoses is *p* < 0.0001. The outlier frequencies (for two standard deviations) in Table 3a are all significantly in excess of that expected due to sampling.

Table 2 shows the number of pathologists by the number of outlier years for two standard deviations. Stated differently, Table 2 is a tabulation of the 105 control charts; the question answered is: how many > 2 SD outliers do each of the 15 pathologists have for a given diagnosis? Fig. 1A shows one of the 8 pathologists that had zero HGD outliers (all circles between the two dashed blue control lines). Figure 1C is one of the two pathologists that had zero SSA outliers. The outliers-years found in Table 1a, are related to the numbers in Table 2; in Table 2 for HGD: 5 pathologists with 1 outlier year each + 2 pathologists with 2 outlier years each = 9 pathologist-year outliers (> 2 SD) in Table 1a. Table 2 shows that there is good self-consistency for HGD; eight pathologists had zero outlier years. It also shows that SSA had marked changes; two pathologists had six outlier years (one of these two pathologist’s normalized PDRs are shown in Fig. 1G).

The data set cleansed of (1) pathologist interpreting < 600 specimens, (2) submitting physicians submitting < 600 specimens, and (3) rare/ambiguous anatomical sites, contained 52,760 colorectal polyp specimens. Two of the 27 pathologists never called SSA; these were non-zeroed to facilitate numerical convergence.

The random effects models (see Table 4) demonstrated that the pathologist, submitting MD, and anatomical location are all strong predictors (*p* < 0.0001) of histomorphologic diagnosis of TA, HGD, TVA + VA, HP, and SSA.

**Table 4:** Generalized linear model results.

Factor	DF	F Value	*p*-value
**Outcome: TA_ad**			
PATHOLOGIST	26	8.34	< .0001
CLINICIAN	45	5.63	< .0001
LOC_FULL_CR	8	646.72	< .0001
YEAR_VAR	6	6.98	< .0001
**Outcome: TVA**			
PATHOLOGIST	26	24.61	< .0001
CLINICIAN	45	7.1	< .0001
LOC_FULL_CR	8	47.52	< .0001
YEAR_VAR	6	12.14	< .0001
**Outcome: HGD**			
PATHOLOGIST	26	5.42	< .0001
CLINICIAN	45	4.26	< .0001
LOC_FULL_CR	8	11.26	< .0001
YEAR_VAR	6	7.33	< .0001
**Outcome: HP**			
PATHOLOGIST	26	21.96	< .0001
CLINICIAN	45	5.49	< .0001
LOC_FULL_CR	8	767.72	< .0001
YEAR_VAR	6	15.27	< .0001
**Outcome: SSA**			
PATHOLOGIST	26	85	< .0001
CLINICIAN	45	2.69	< .0001
LOC_FULL_CR	8	161.75	< .0001
YEAR_VAR	6	60.09	< .0001

“PATHOLOGIST” and “CLINICIAN” are variables that represent individual pathologists and individual submitting physicians/surgeons. “LOC_FULL_CR” is a variable that reperesent the anatomical location; it can be one of nine locations in the colon/rectum (rectum, rectosigmoid colon, sigmoid colon, descending colon, splenic flexure of colon, transverse colon, hepatic flexure of colon, ascending colon, cecum). “YEAR_VAR” is the year in which the specimen was accessioned. “DF” is the degrees of freedom. Tubular adenoma and adenoma NOS (TA_ad) were lumped in this analysis, as a subset of pathologists (early in the study period) signed cases as “adenoma” without further specifying. These are presumed to represent tubular adenomas.

## Discussion

The HFTSMA algorithm appears to deliver reliable categorizations that are. sufficient to assess diagnostic variances on the order of 1%. Non-categorized polyps appear to represent a separate group/set of diagnoses that are predominantly descriptive diagnoses or ambiguously-worded reports that cannot be easily classified.

Most of the pathologists in the cohort had relatively stable diagnostic rates over time, but there were apparent outliers. The relative uniformity in some diagnoses (e.g. high-grade dysplasia) provides good evidence against the presence of case assignment bias.

The hospital sites show some clustering of patterns in PDR. This may be mostly explained by the presence of group set-point bias rather than true differences between hospital sites.

The “clinicians” factor (submitting MD) appears to explain less variation in the data than the “pathologist” factor.

Traditional inter-rater studies look at a relatively small set of cases and rarely examine diagnostic bias over a longer period of time. This study examined the reports in an entire region over a period of seven years.

While high-grade dysplasia and villous component are predictive of neoplasia risk in large cohorts, the findings herein suggest risk stratification using high-grade dysplasia and villous component suboptimally risk-stratifies individual patients, due to the consistent (presumptive substantial inter-rater) variation in the pathologist diagnostic rate.

Generally, the findings demonstrate that the histomorphologic interpretation of colorectal polyps could be less varied than seen herein, and imply that (statistical) process control (or an automated analysis), that reproduces the categorization biases of one pathologist (or a panel of pathologists), would deliver more uniformity.

Based on our prior work^11^ and PDR data (predominantly published as conference abstracts), we are not convinced that more sub-specialization is the only answer. We also note that disagreement among subspecialists may be very high^12^. The processes changes (independent of training) may significantly improve quality^13,14^.

### Limitations

A few pathologists moved between hospital sites in the 7-year period; however, none of the 15 pathologists interpreted less than 92% of their specimens from their primary site. This is a confounder that was not specifically controlled for in the construction of the control charts; however, the effect is suspected to be small.

It is not possible to determine the ideal rate(s) in this study. Whether large true differences exist between the hospital sites cannot be determined within the context of this study. It is possible that the differences between the hospital sites is totally or partially explained by group bias. A significant number of specimens (~ 300 from each hospital site) would need to be reviewed by an expert panel, as the differences are likely to be small. Normalized plots showing the polyps by lumped anatomical site (left colon, mid colon, right colon) and pathologist suggest there may be differences between the hospitals (see supplemental materials).

Based on how the specimens are submitted and reported in routine practice, it is not possible to do the analysis on the level of the individual polyp.

We did not attempt to make control charts based on the yearly rates for each hospital, as the study set (15 pathologists with data over all years) was deemed to be too small to sub-stratify. This true limitation was explored with the random effects model and logistic regression.

Significant changes over time were identified with the random effect model, thus calling into question the “disease stability” assumption that is a part of the control chart analysis. We are not convinced these changes affect the overall conclusions due to the variation seen in the control charts. It is impossible to determine whether the change over time is (1) diagnostic re-calibration/diagnostic drift by selected pathologists; (2) a change in the population or (3) some combination of drift and population change. The trend data suggests strongly that there is re-calibration. We suspect there was a shift between TA and HP in the population. Supplemental materials show how the diagnoses varied over the seven-year period. There are very clear trends in HP and SSA, which may be rationalized in the context of when SSA was described, and by knowledge dissemination rates in medicine.

Specific healthcare provider characteristics (e.g. training, years in practice, type of practice) were not collected as part of this study. These may be significant predictors.

The study is observational and the data collected is influenced to certain extent by conscious changes to clinical practice. A subgroup of pathologists (due to a quality improvement project/pilot study^15^ were aware of their diagnostic rates in the last two years of the study period and a subset of those adjusted their practice. This likely decreased consistency with self and thus somewhat decreased the study’s effect size. It is not possible to fully analyze the effect of the subgroup (due the anonymity constraint in the study); however, the control charts show diagnostic rate changes in the early part of the study that are similar in magnitude to changes in the later part of the study period; thus, the overall conclusions are likely unaffected.

### Diagnostic rate awareness and improvement

Colorectal polyps are specimens that may be infrequently reviewed at consensus rounds in relation to their volume; thus, call rate harmonization/calibration that takes place within pathology practices may not occur for these specimens. Also, random case reviews are not powered to detect modest call rate differences and would be prohibitively expensive if powered to do so.

SPC is a mechanism that may facilitate greater uniformity in reporting practices through greater dialogue about true population rates (with ideal pathological interpretation), and promote continuous review of outcome data. In the presence of significant differences in interpretation (that are unlikely to result from case assignment/sampling), suboptimal interpretations may be suspected and a process of resolution implemented through consensus guided by outcome data.

Based on a pilot study of ~ 7054 colorectal polyp specimens (interpreted by 9 pathologists (each year) over two years—Sep 2015–Aug 2017) in conjunction with (1) informing each pathologist of their (TA, HP, SSA, TVA + VA) diagnostic rates, and (2) a group review of (SSA cases) with a gastrointestinal pathology expert, it is possible to increase uniformity in sessile serrated adenoma (SSA) diagnostic rates^15^.We suspect that this process (statistical process control) could be applied more broadly and would lead to further improvements.

## Conclusions

Current diagnostic processes for colorectal polyp specimens leave significant room for further improvement. This work suggests that most pathologists have diagnostic rate stability, and that non-stable rates are likely (conscious or unconscious) practice changes.

Statistical process control (SPC) could result in significantly more uniformity, given that many pathologists have moderate diagnostic rate stability. Thus, the further implementation of SPC in pathology should be pursued, as it could substantially optimize and improve care.

### Supplementary Information

Supplementary Information.

## Data Availability

The data sets generated and/or analyzed during the current study are not publicly available due confidentially reasons but aggregate data is available from the corresponding author on request.

## Appendix A (Diagnostic Codes and Search Strings)

**Table:**

Dx code	String
dx1	Suspicious for adenocarcinoma
dx1	Cannot exclude adenocarcinoma
dx1	Adenocarcinoma can't excluded
dx1	Suspicious for invasive adenocarcinoma
dx2	Invasive adenocarcinoma
dx2	Adenocarcinoma
dx2	Invasive carcinoma
dx3	Intramucosal adenocarcinoma
dx3	Of high-grade dysplasia
dx3	Of high grade dysplasia
dx3	With high-grade dysplasia
dx3	With high grade dysplasia
dx3	Focal high grade dysplasia
dx3	Focal high-grade dysplasia
dx3	Showing high grade dysplasia
dx3	Showing high-grade dysplasia
dx4	Suspicious for lymphoma
dx4	Atypical lymphoid proliferation
dx5	MALT lymphoma
dx5	Mantle cell lymphoma
dx6	Tubular adenoma
dx6	Tubular adenomata
dx7	Hyperplastic polyp
dx7	Hyperplastic polyp
dx8	Hyperplastic changes
dx8	Hyperplastic mucosal changes
dx8	Hyperplastic features
dx8	Hyperplastic-like features
dx9	Tubulovillous adenoma
dx9	Tubularvillous adenoma
dx9	Tubulo-villous adenoma
dx9	Villotubular adenoma
dx10	Villous adenoma
dx11	Sessile serrated adenoma
dx11	Sessile serrated polyp
dx11	Serrated sessile adenoma
dx11	Serrated polyp, favor adenoma
dx11	Serrated polyp, favour adenoma
dx11	Serrated polyps, favor adenomata
dx12	Traditional serrated adenoma
dx13	Serrated adenoma
dx13	Serrated adenomata
dx13	Serrated polyp
dx14	No pathologic finding
dx14	Unremarkable fragment of large bowel mucosa
dx14	Unremarkable large bowel mucosa
dx14	Benign colonic mucosa
dx14	Benign large bowel mucosa
dx14	Normal colonic mucosa
dx14	NO DIAGNOSTIC ABNORMALITY
dx14	Unremarkable colonic mucosa
dx14	Mucosa without significant pathology
dx14	Mucosa within normal limits
dx14	No specific pathology
dx14	No significant histopathologic abnormality
dx14	NEGATIVE for evidence of significant pathology
dx14	No significant pathological changes
dx14	No pathological changes
dx14	No evidence of polyp
dx14	Polypoid mucosa
dx14	Colonic mucosa with prominent lymphoid follicles
dx14	Negative for apparent pathology
dx14	No pathological diagnosis
dx14	CAUTERY ARTIFACT, NOT FURTHER DIAGNOSTIC
dx14	Reactive changes, NEGATIVE
dx14	Colonic mucosa with lymphoid aggregate
dx14	Large bowel mucosa with no definite polyp
dx14	Non-diagnostic polypoid colonic mucosa
dx14	Bowel mucosa with no significant findings
dx14	Bowel mucosa with no evidence of polyp
dx14	No polyp
dx14	No specific polyp
dx14	No definitive polyp
dx14	No definite polyp
dx14	No specific pathologic diagnosis
dx14	No significant pathology identified
dx14	No significant pathological abnormalities
dx14	No findings
dx14	No histopathological abnormality
dx14	Prominent mucosal folds
dx14	Mucosa, likely mucosal fold
dx14	Without diagnostic abnormality
dx14	Unremarkable mucosal tissue
dx14	No significant findings
dx14	Colonic mucosa with no pathology
dx14	No significant inflammation or other findings
dx15	POLYPOID COLONIC MUCOSA
dx15	Prominent lymphoid aggregate
dx15	Lymphoid aggregate
dx15	Large intestinal mucosa slightly polypoid with lymphoid aggregates
dx15	Mucosa with lympho-follicular hyperplasia
dx15	Lymphoid follicle
dx15	Benign lymphoid aggregate
dx15	Mucosal germinal centre
dx15	Lymphoid hyperplasia
dx15	Lymphocytic aggregates
dx16	Inflammatory polyp
dx16	Inflammatory pseudopolyp
dx16	Inflammatory large bowel polyp
dx16	Inflammatory-type polyp
dx16	Inflamed polyp
dx17	Hamartomatous polyp
dx18	Granulation tissue
dx19	Active colitis
dx19	Active proctitis
dx19	Acute cryptitis
dx20	Poorly preserved colonic mucosa
dx21	Solitary rectal ulcer
dx21	Mucosal prolapse syndrome
dx21	Mucosal Prolapse
dx21	Mucosal prolapse-like polyp
dx21	Mucosa with prolapse like changes
dx22	Melanosis coli
dx22	Pseudomelanosis coli
dx22	Slight melanosis
dx23	Juvenile polyp
dx23	Juvenile type polyp
dx23	Retension polyp
dx24	Lipoma
dx25	Granular cell tumour
dx25	Granular cell tumor
dx26	Ischemic colitis
dx27	Leiomyoma
dx28	Xanthoma
dx28	Xanthoma/xanthelasma
dx29	Hemorrhoid
dx30	Prolapse changes
dx31	Fecal material
dx31	Fecal matter only
dx31	Vegetable fibres
dx31	Vegetable matter
dx31	Fecal matter
dx31	Feces
dx31	Food material
dx31	Polypoid vegetable resembling seed
dx31	Degenerated meat fibres
dx32	NEGATIVE for dysplasia
dx32	NEGATIVE for evidence of dysplasia
dx32	No neoplasia present
dx32	Negative for conventional/adenomatous dysplasia
dx32	Negative for adenomatous polyp or dysplasia
dx32	Negative for adenoma
dx33	Tissue not identified
dx33	No tissue is identified
dx33	No tissue present
dx33	No tissue was found
dx33	No microscopic assessment possible
dx33	Tissue did not survive processing
dx33	Did not survive tissue processing
dx33	No material present after processing
dx33	See gross
dx33	No specimen received
dx33	No specimen identified
dx33	Insufficient for evaluation
dx33	Insufficient for assessment
dx33	Insufficient tissue for histologic assessment
dx33	No colon tissue is observed
dx33	Mucosa, not diagnostic
dx34	Negative for high grade dysplasia
dx34	Negative for high-grade dysplasia
dx34	No evidence of high grade dysplasia
dx34	No evidence of high-grade dysplasia
dx34	No evidence of high dysplasia
dx34	No definite evidence of high-grade dysplasia
dx34	No convincing evidence of high grade dysplasia
dx34	Without high grade dysplasia
dx34	Without high-grade dysplasia
dx35	NEGATIVE FOR DYSPLASIA OR MALIGNANCY
dx35	Negative for high-grade dysplasia and malignancy
dx35	Negative for high grade dysplasia and malignancy
dx35	Negative for high-grade dysplasia or invasive malignancy
dx35	Negative for high grade dysplasia or invasive malignancy
dx35	Negative for high-grade dysplasia or invasive carcinoma
dx35	Negative for high grade dysplasia or invasive carcinoma
dx35	Negative for high-grade dysplasia and invasive carcinoma
dx35	Negative for high grade dysplasia and invasive carcinoma
dx35	No convincing evidence of high grade dysplasia or adenocarcinoma
dx36	Cautery/crush artifact
dx36	Cautery artifact
dx36	Cautery artefact
dx36	Cauterized tissue
dx36	Cauterized colonic mucosa
dx36	Polypoid cauterized mucosa
dx36	Crushed fragments of large bowel
dx37	Focal adenomatous changes
dx37	Focal adenomatous change
dx37	Fragments of adenoma
dx37	Possible adenomatous change
dx37	Adenoma
dx37	Suspicious for Adenomatous Changes
dx37	Adenomatous change
dx37	Adenomatous mucosal change
dx37	-ADENOMA(S)
dx38	Chronic inflammation
dx38	Chronic inflammation only
dx39	Dysplasia associated lesion or mass
dx39	Features of DALM
dx40	Carcinoid
dx40	Neuroendocrine tumour
dx40	Neuroendocrine tumor

## Appendix B (Location Codes, Search Strings and Distances)

The location code was based on the location provided in the “source of specimen” section of the report. If the location was given in centimetres from the anal verge, it was converted to named location, based on approximate measures used by NCI.

### Appendix B-1: Codes and location dictionary

**Table:**

Location code	String
loc1	Rectal
loc1	Rectum
loc2	Rectosigmoid
loc2	Rectum—sigmoid
loc2	Rectal—sigmoid
loc3	Sigmoid
loc4	Descending
loc5	Splenic
loc6	Transverse
loc7	Hepatic
loc8	Ascending
loc9	Cecum
loc9	Cecal
loc9	Appendiceal orifice
loc10	Left colon
loc11	Right colon
loc12	Anastomosis
loc12	Anastomotic

### Appendix B-2: Codes and location table

**Table:**

Location code	Distance (cm)
loc1	< 13
loc2	< 18, ≥ 13
loc3	< 58, ≥ 18
loc4	< 79, ≥ 58
loc5	< 84, ≥ 79
loc6	< 130, ≥ 84
loc7	< 136, ≥ 130
loc8	< 147, ≥ 136
loc9	< 151, ≥ 147

## Appendix C (Control charts/normalized funnel plots)

Based on the normal approximation of the binomial distribution:

1 $${\text{SE}} = \sqrt {\frac{{i \times (1 - i)}}{n}}$$

where:

SD = standard deviation.

i = ideal (diagnostic) rate †

n = number of specimens interpreted.

† The ideal rate in this study is approximated by the group median rate.

The healthcare provider rate (pathologist diagnostic rate) is normalized as follows:

2 $$N_{j} = \frac{{M_{j} - i}}{{{\text{SE}}_{j} }}$$

where:

N_j_ = healthcare provider rate for healthcare provider “j”.

M_j_ = measured rate for the healthcare provider “j”.

i = ideal (diagnostic) rate.

SD_j_ = SD for healthcare provider “j”.

Equation (2) can be substituted into Eq. (1):

3 $$M_{j} - N_{j} \left[ {\sqrt[i]{{\frac{(1 - i)}{n}}}} \right] + {\text{i}}$$

To normalize we presume that the “SD” is equivalent and that only “n” changes. This amounts to forming two equations from Eq. 3 and solving for the normed M_j_. After some rearrangement one can derive a conversion equation:

4 $$M_{{{\text{jnormed}}}} = \left[ {M_{{{\text{jmeasured}}}} - i} \right]\frac{{\sqrt[i]{{\frac{(1 - i)}{{n_{{{\text{jnormed}}}} }}}}}}{{\sqrt[i]{{\frac{(1 - i)}{{n_{{{\text{jmeasured}}}} }}}}}} + {\text{i}}$$

where

M_j normed_ = normed (diagnostic) rate for healthcare provider “j”.

M_j measured_ = measured (diagnostic) rate for healthcare provider “j”.

n_j normed_ = normed number of specimens handled (interpreted) by healthcare provider “j”.

n_j measured_ = number of specimens handled (interpreted) by healthcare provider “j”.

i = ideal (diagnostic) rate.

## Appendix D (Probability Calculation)

The probability (Y) of n and < n outliers in k samples is dependent on the probability of the individual outlier (p), and the binomial cumulative distribution function:

5 $${\text{Y }} = {\text{ binocdf}}\left( {{\text{n}},{\text{ k}},{\text{ p}}} \right)$$

The probability (X) of n and > n outliers in this context is the complement of ‘n-1’:

6 $${\text{X }} = { 1} - {\text{ binocdf }}\left( {{\text{n}} - {1},{\text{ k}},{\text{ p}}} \right)$$

## Appendix E: Understanding statistical process control

### Overview

Statistical process control is a cyclical process that involves:

1. Repeated measurement
2. Assessment of the variation in the measurement (using statistics)
3. Possible adjustments in the process to ensure that future measurements fall within a prescribed range, as defined by the so-called “control lines”

### Statistical process control applied to diagnostic pathology at a conceptual level

In the diagnostic pathology context—if all the following are true:

1. Cases are assigned randomly to pathologists from a given population
2. Pathologists see large numbers of a particular type of case (e.g. 200 cases)

Then:

It is likely that the diagnostic rate (of say ‘tubular adenoma’) is similar for different pathologists (e.g. Pathologist ‘A’ diagnoses 102 tubular adenoma in 200 cases, Pathologist ‘B’ diagnosies 105 tubular adenomas in 200 cases) ****

**** Statistically, it can be stated that there is range in which the diagnostic rate (number of diagnoses/total cases interpreted) will fall 95% of the time.

If pathologists have significantly different diagnostic rates:

It is likely that they interpret cases differently and it may be possible to reduce diagnostic variation, via diagnostic calibration.

### Diagnostic calibration is not new

Pathologists can change their diagnostic rates over time and may do this when (1) their cases are reviewed and found discrepant, (2) they review cases of (trusted) other (more experienced or subspeciality trained) pathologists, and (3) when new diagnostic entities are discovered or diagnostic criteria revised.

If a pathologist consistently (over time) has a diagnostic rate for tubular adenoma (e.g. 20/200 = 0.2) that is outside the range expected (in relation to the diagnostic rates of all their colleagues (~ 100/200 = 0.5)), one can infer that it would be possible to “re-train” that individual (or all the other pathologists) to arrive at the same diagnostic rate.

SPC in a nutshell is: *systematically looking at the data (with statistics) to calibrate a process*; in anatomical pathology it would be a process to look at diagnostic rates and feed those diagnostic rates back to the pathologists—such that they can adjust to a target rate/find agreement on what the target rate should be.

### Statistical process control is predicated on two conditions

Condition (1) the process that one wants to control is stable over time (such that it is possible to predict the future) or can be made stable.

Condition (2) there is an ability to adjust the control variable * in a meaningful way—in relation to the control lines **.

* a c*ontrol variable* is: a parameter that one wants to control, e.g. the diagnostic rate of ‘sessile serrated adenoma’.

** *control lines* in SPC are determined by the “expected” statistical variation—when the process is in control/running optimally, e.g. the control parameter falls within a given range 95% of the times. Control limits are confidence intervals and are directly analogous to the funnel lines on funnel plots.

“Ability to adjust the control variable in a meaningful way” implies the following:

1. the variation (between providers) one expects to see due to chance is smaller than the (actual) variation that is observed; this implies that improvement is possible ***
2. an intervention can change the control variable in a substantive way, such that the variation is reduced

*** If the variation is less than the variation by chance the process is in control (or one may need a larger sample size).

### The conditions for statistical process control and the objective of the manuscript

‘Condition 1’ for SPC is met if there is *diagnostic stability*.

‘Condition 2’ for SPC is met if there is *significant diagnostic variation—that is stable (e.g. one pathologist is a consistent outlier in relation to the median diagnostic rate *****),* and it is assumed that pathologists want to improve their practice/can be encouraged to make positive changes (see section “*Diagnostic Calibration is Not New*”).

‘Condition 1’ and ‘Condition 2’ are sufficient to infer that SPC should be feasible and could be used to improve care.

***** It should be noted that: the ‘median diagnostic rate’ may not be the ideal diagnostic rate for a given population. It is possible that an ‘outlier’ pathologist represents the ideal diagnostic rate.

In SPC, one talks of variation due to an “assignable cause” [a modifiable factor] and “common cause” [un-modifiable factors]. In the language of SPC, the question succinctly is: *Is the pathologist an assignable cause?*

If diagnostic rates are stable [Condition 1], and the pathologist is an “assignable cause” [Condition 2], SPC should be feasible.
