# Supplementary material for: Stability of diagnostic rate in a cohort of 38,813 colorectal polyp specimens and implications for histomorphology and statistical process control
Source: Sci Rep. 2021 Aug 20;11:16942. doi: 10.1038/s41598-021-95862-2 (PMC8379162; doi:10.1038/s41598-021-95862-2)
Supplement: Supplementary file 1 — Supplementary Information. [file 41598_2021_95862_MOESM1_ESM.pdf]

Supplemental Figures and Tables

for

Stability of Diagnostic Rate in a Cohort of 38,813 Colorectal Polyp Specimens and Implications for Histomorphology and Statistical Process Control

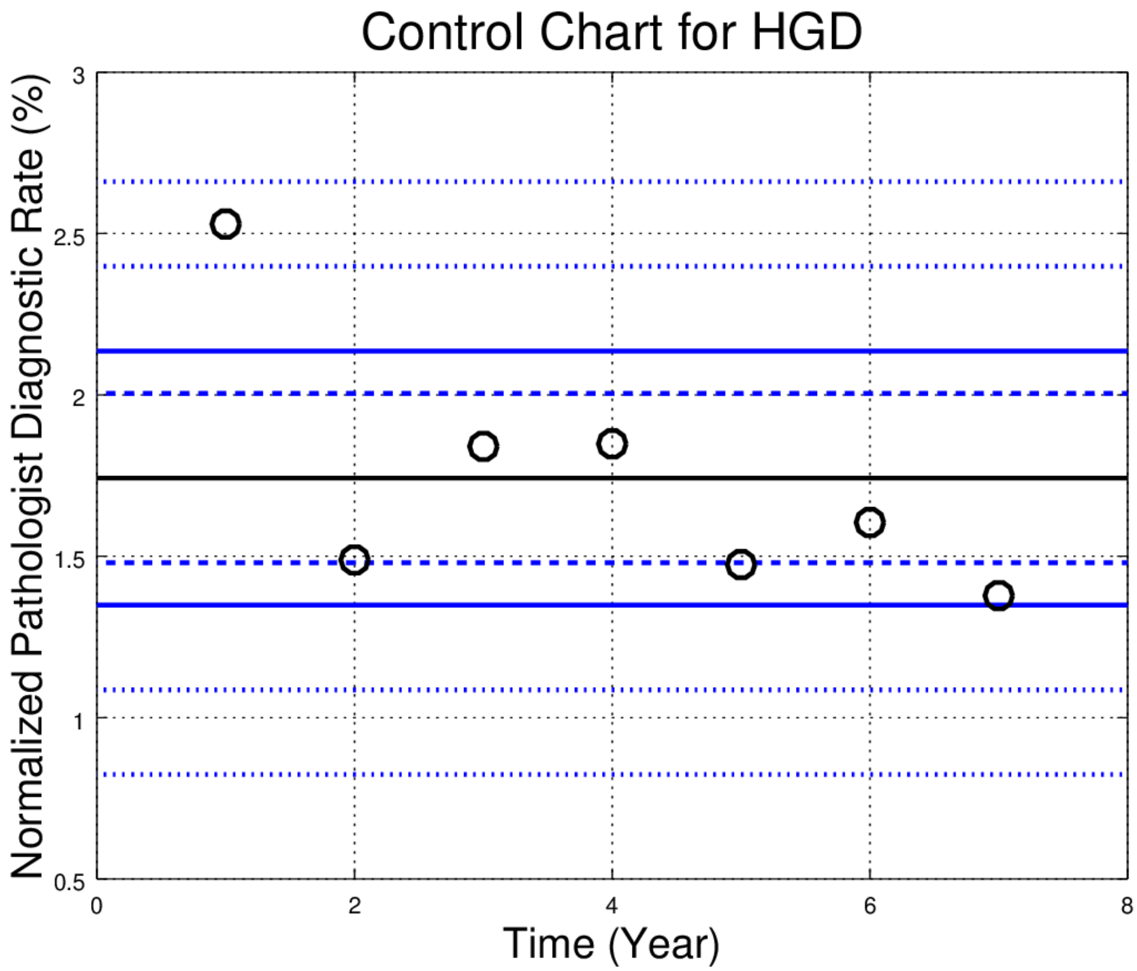

Supplemental Figure A: Mean call rate of high-grade dysplasia (HGD) for each year of the study. The control lines are centred on the average call rate over the seven years of the study.

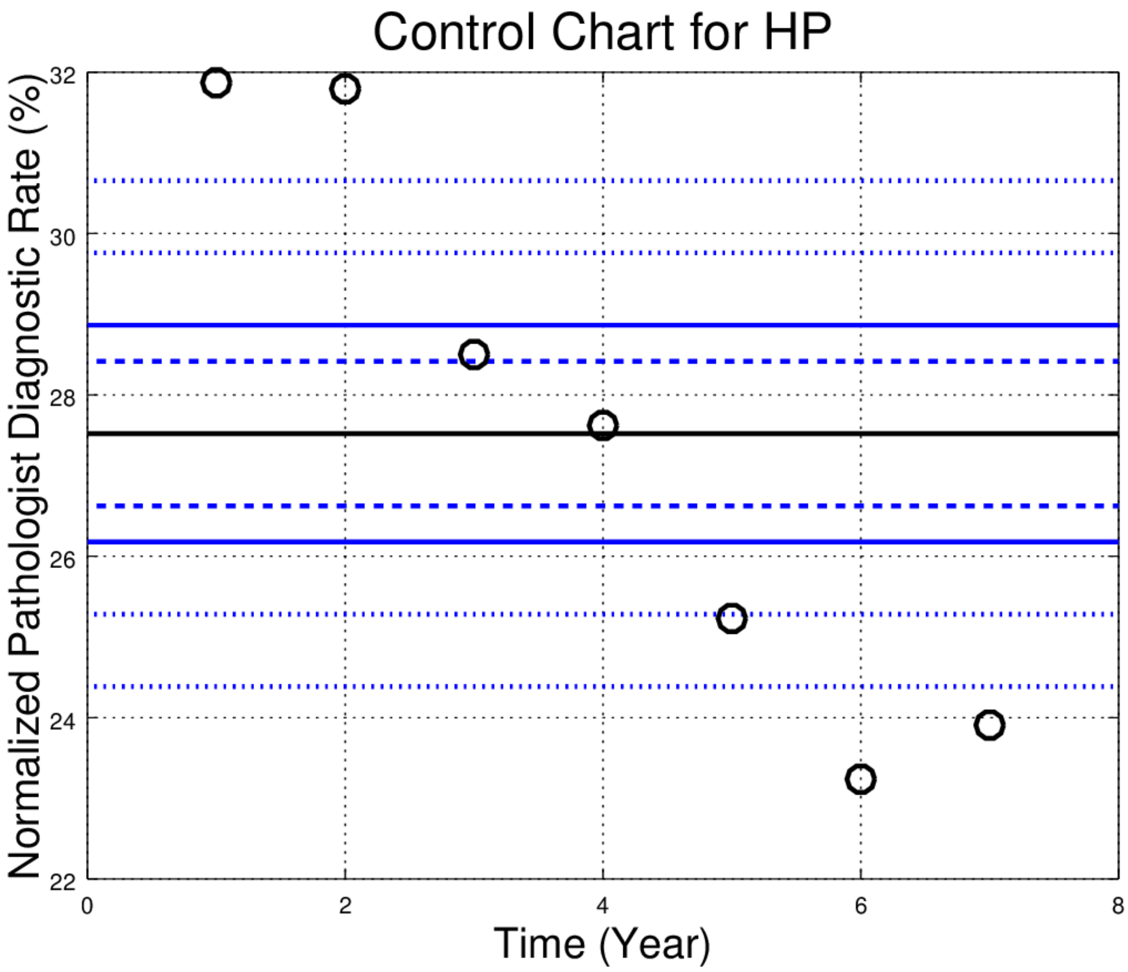

Supplemental Figure B: Mean call rate of hyperplastic polyp (HP) for each year of the study. The control lines are centred on the average call rate over the seven years of the study.

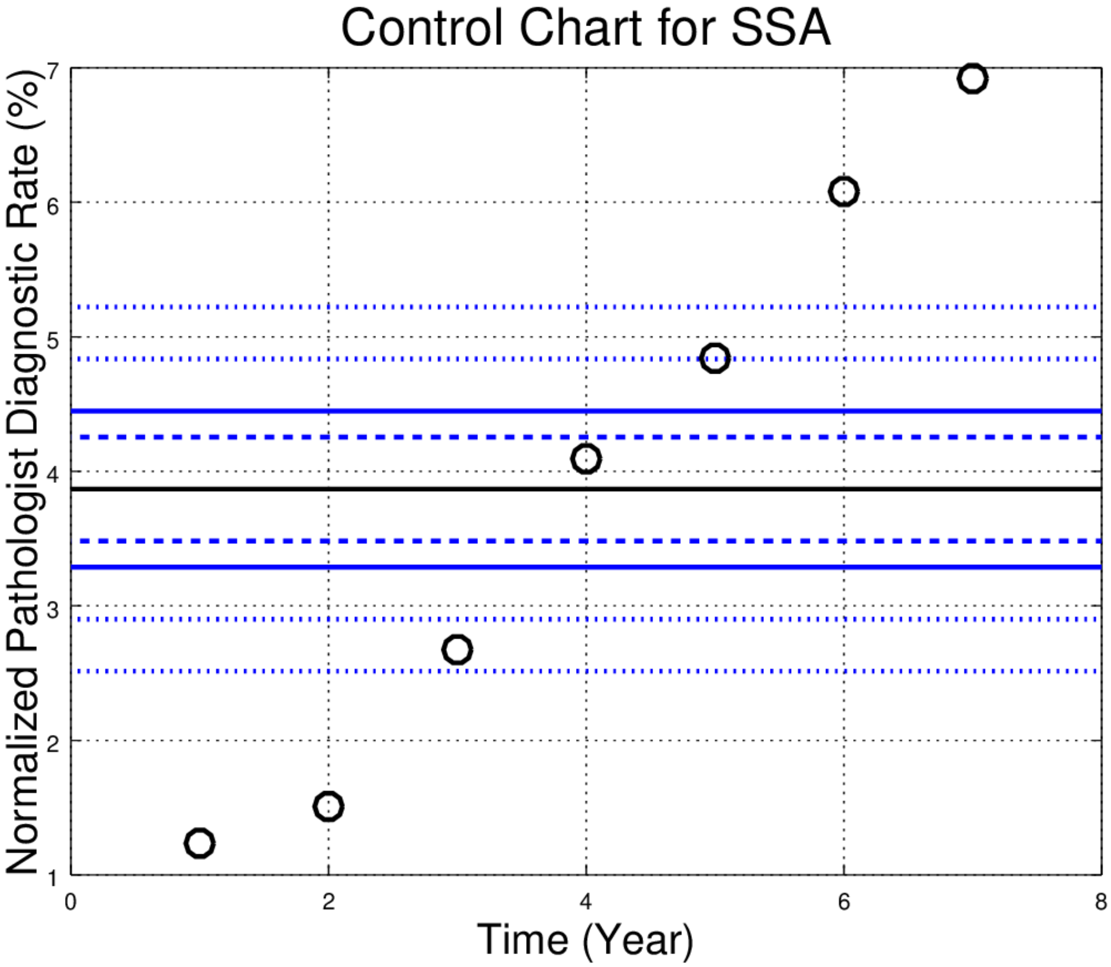

Supplemental Figure C: Mean call rate of sessile serrated adenoma (SSA) for each year of the study. The control lines are centred on the average call rate over the seven years of the study.

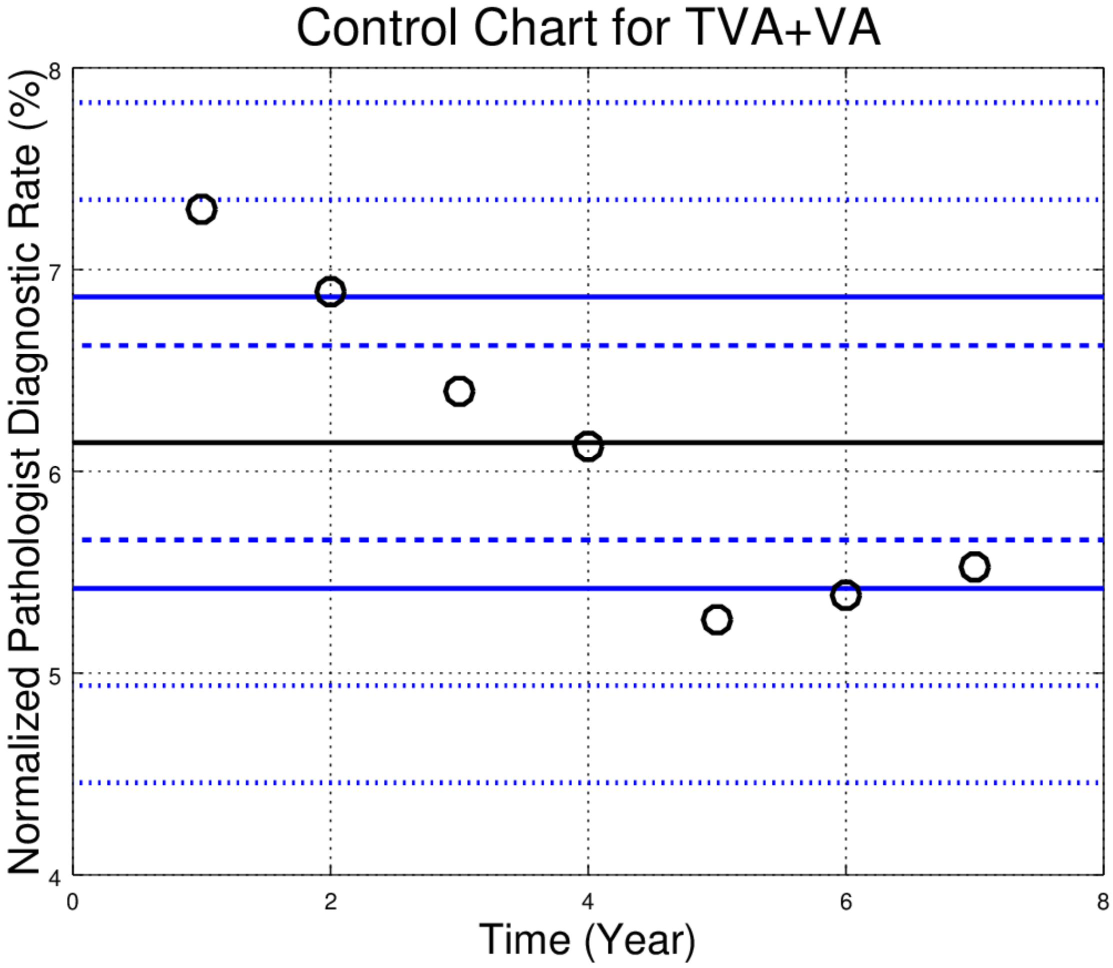

Supplemental Figure D: Mean call rate of tubulovillous adenoma+villous adenoma (TVA+VA) for each year of the study. The control lines are centred on the average call rate over the seven years of the study.

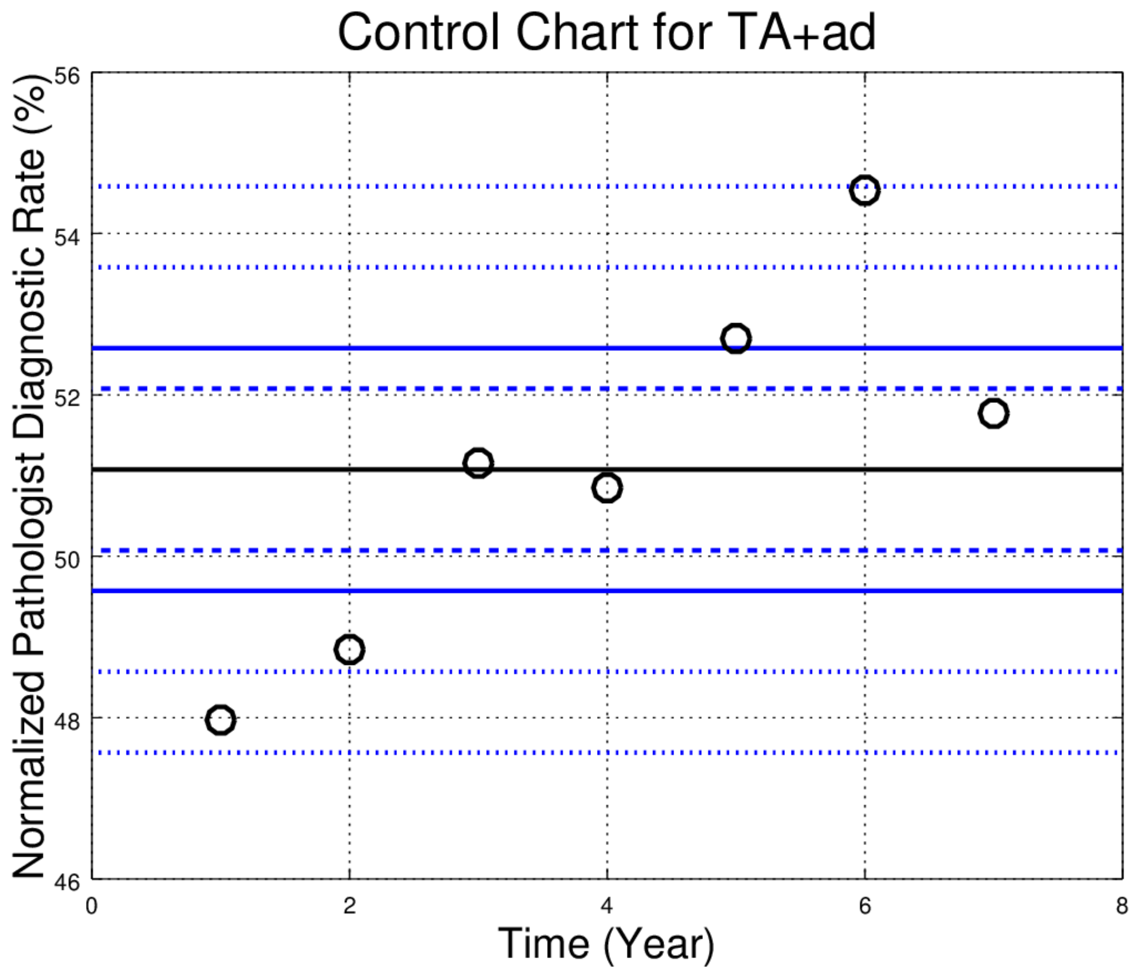

Supplemental Figure E: Mean call rate of tubular adenoma+adenoma not otherwise specified (TA+ad) for each year of the study. The control lines are centred on the average call rate over the seven years of the study.

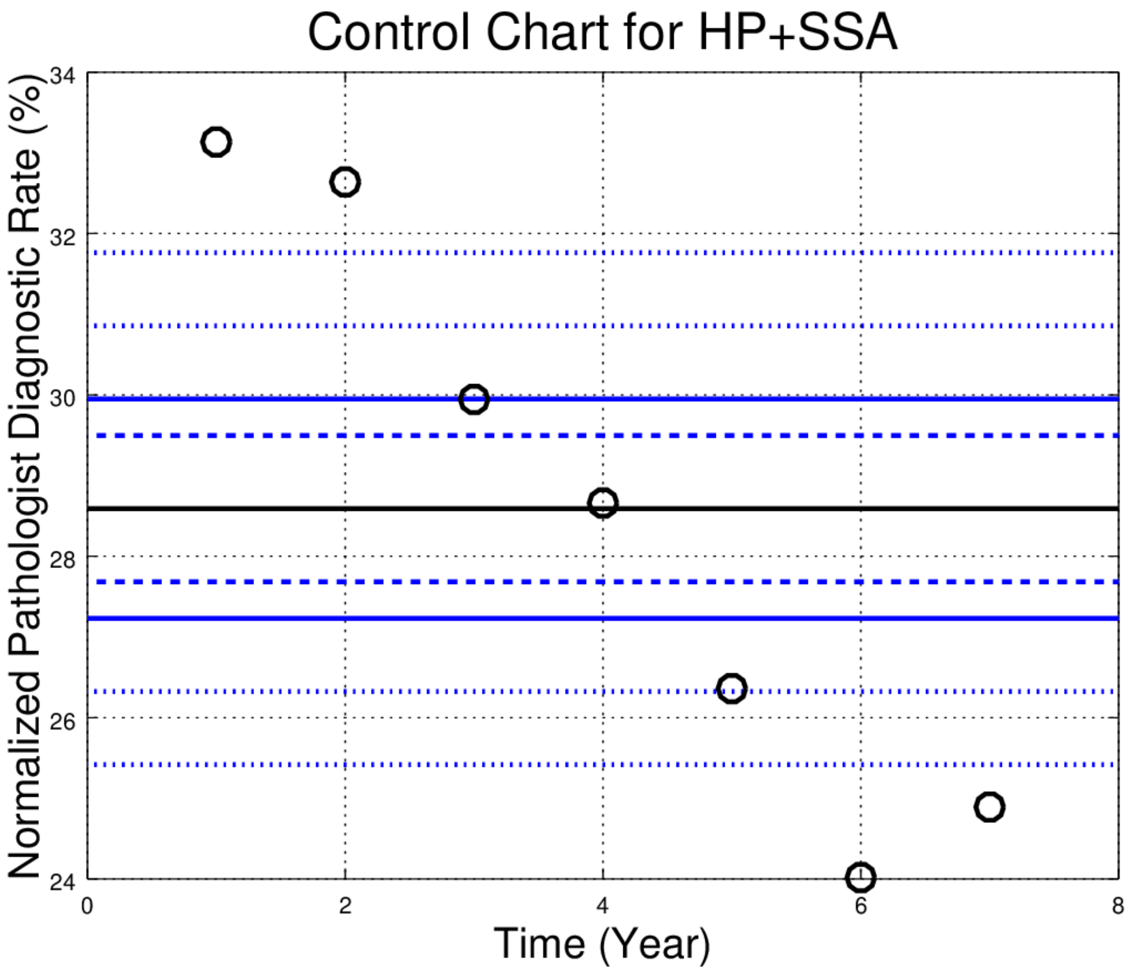

Supplemental Figure F: Mean call rate of hyperplastic polyp+sessile serrated adenoma (HP+SSA) for each year of the study. The control lines are centred on the average call rate over the seven years of the study.

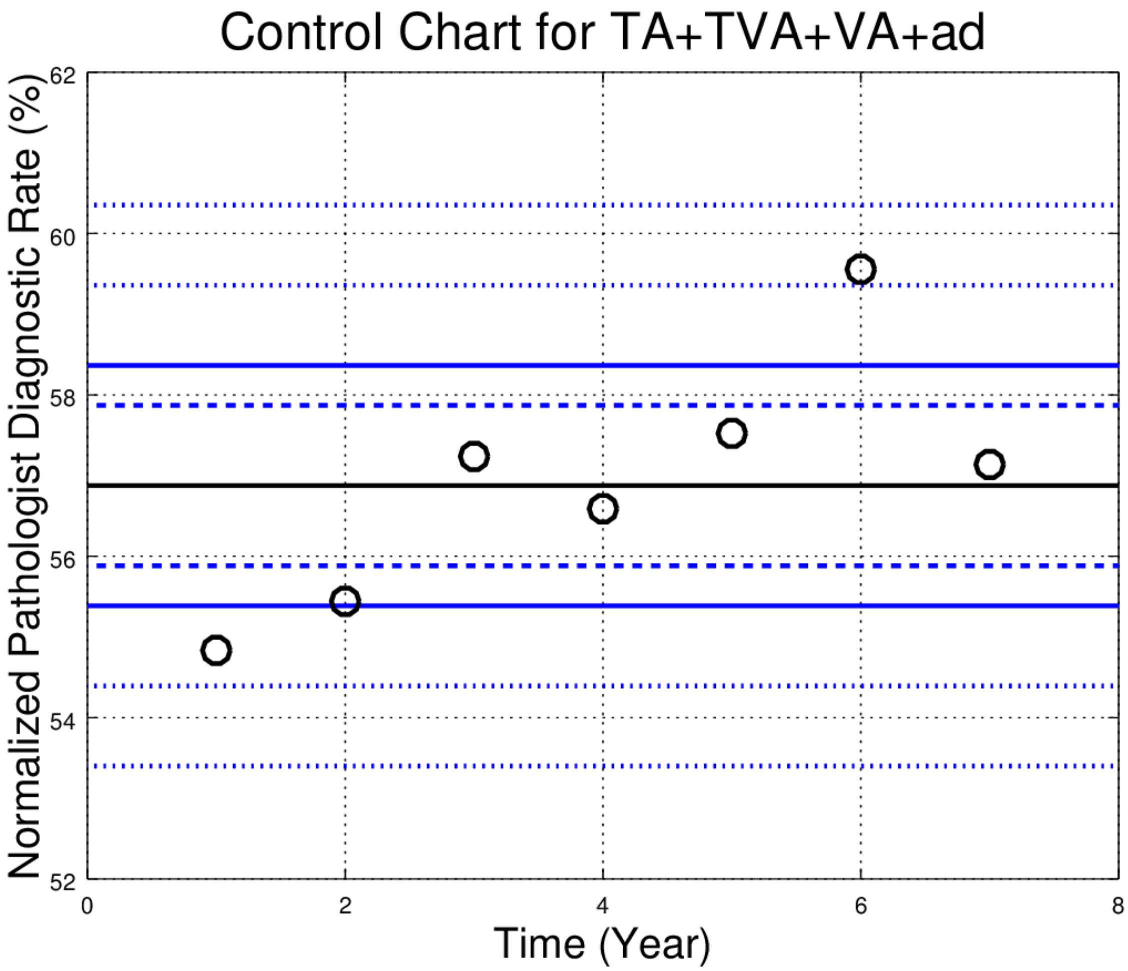

Supplemental Figure G: Mean call rate of tubular adenoma+tubulovillous adenoma+villous adenoma+adenoma not otherwise specified (TA+TVA+VA+ad) for each year of the study. The control lines are centred on the average call rate over the seven years of the study.

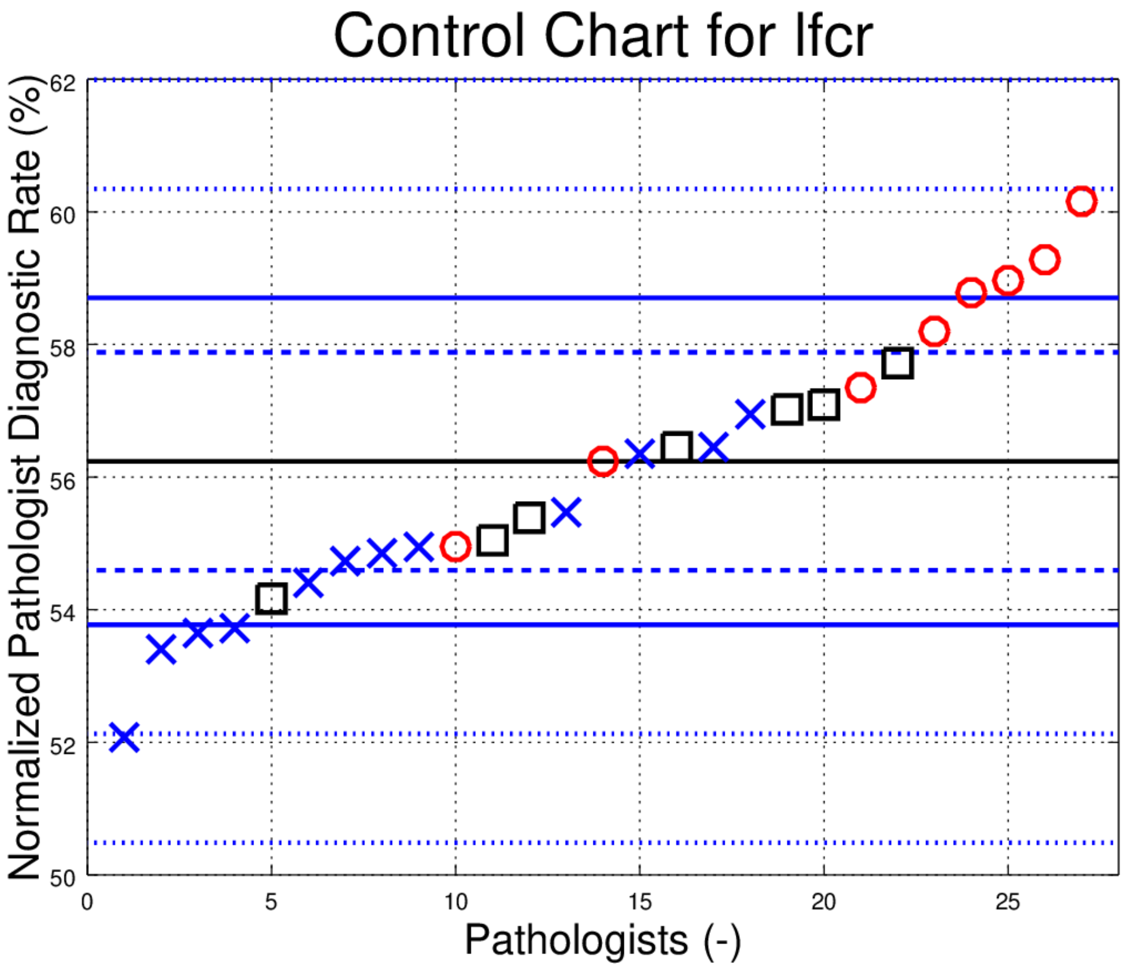

Supplemental Figure H: Normalized left colon/rectum cases by pathologist. The control lines are centred on the average rate over the seven years of the study. This data is not influenced by pathologist interpretation; it is based only on what was on the pathology requisition.

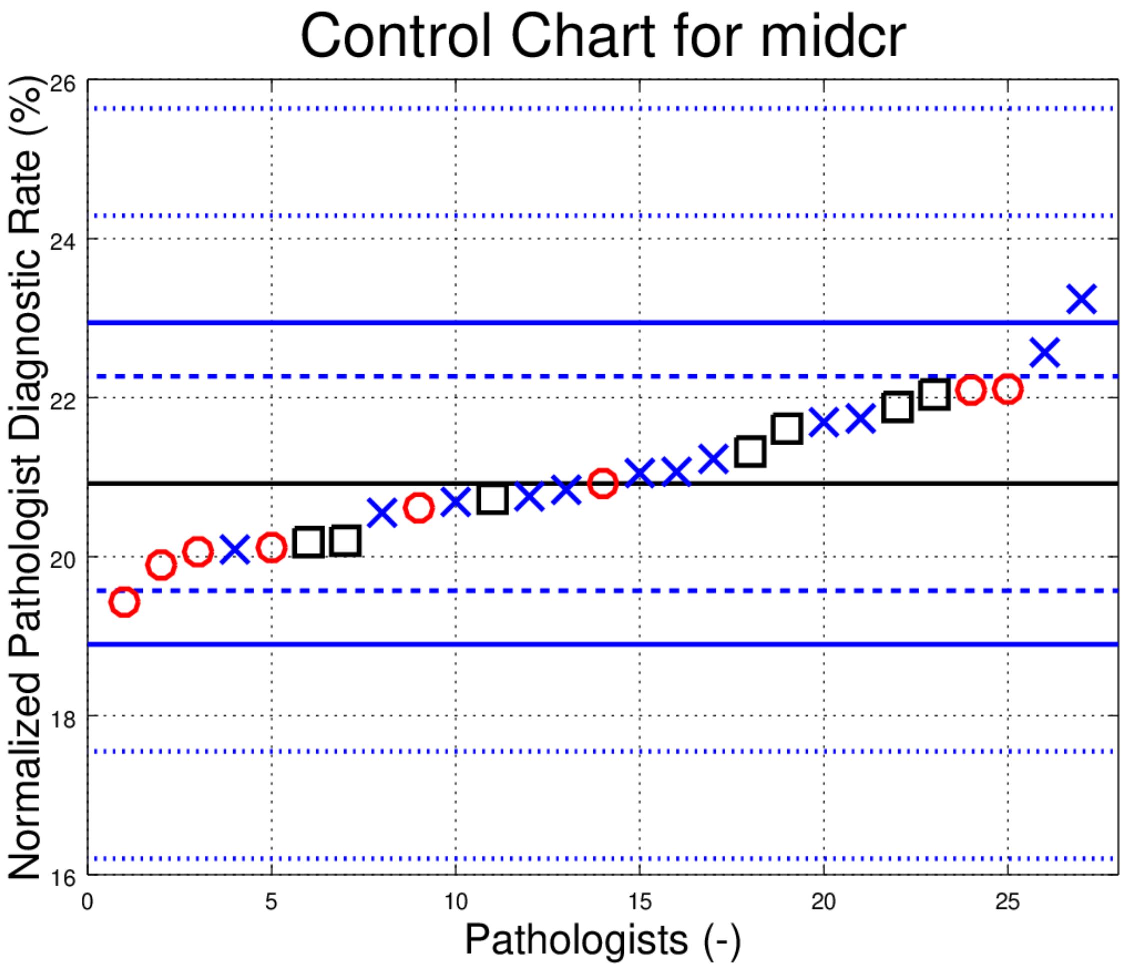

Supplemental Figure I: Normalized mid colon cases by pathologist; mid colon includes splenic flexure, transverse colon and hepatic flexure. The control lines are centred on the average rate over the seven years of the study. This data is not influenced by pathologist interpretation; it is based only on what was on the pathology requisition.

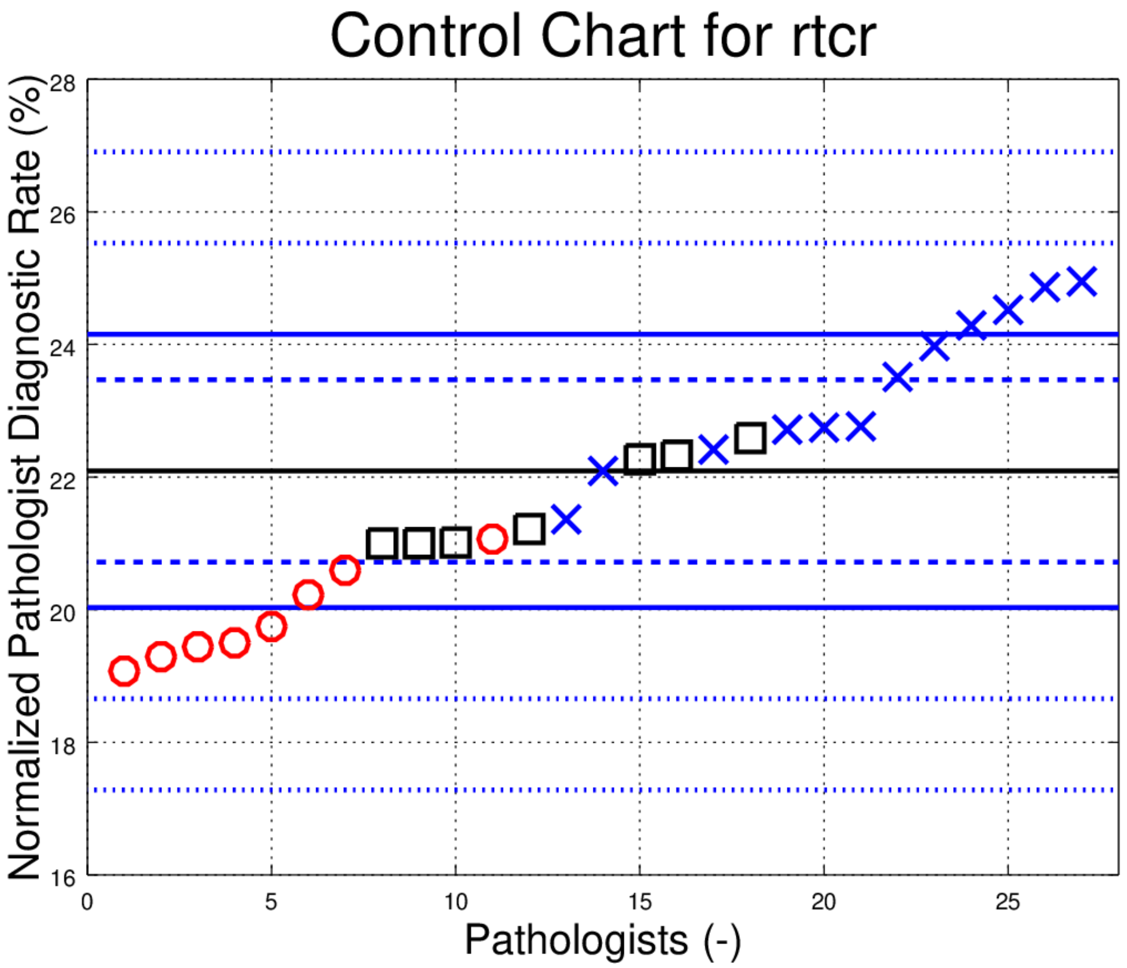

Supplemental Figure J: Normalized right colon cases by pathologist. The control lines are centred on the average rate over the seven years of the study. This data is not influenced by pathologist interpretation; it is based only on what was on the pathology requisition.

Table S1: Colorectal Polyp Specimens – Cohort Overview

Diagnosis	Number	Percent
High-grade dysplasia (HGD)	1117	1.7
Tubular adenoma (TA)	30215	47.1
Hyperplastic polyp (HP)	17645	27.5
Tubulovillous adenoma (TVA)	3561	5.6
Villous adenoma (VA)	384	0.6
Sessile serrated adenoma (SSA)	2480	3.9
Adenoma NOS	3216	5.0
Traditional serrated adenoma (TSA)	101	0.2
Adenocarinoma	296	0.5
**Lumped categories**		
TA + adenoma NOS (TA_ad)	33431	52.1
TVA + VA	3945	6.2
Negative for pathology	5424	8.5
**Total specimens**	**64115**	**100.0**

Table S1: Overview of colorectal polyp specimens, selected categories. NOS = not otherwise specified. It should be noted that the diagnoses are not mutually exclusive as several polyps can be submitted within the same container

Table S2: Fraction that are Outliers by Self, Site, and all 27 Pathologists

Variation	To self HGD	To site HGD	To all 27 HGD
**<2 SD**	0.91	0.59	0.41
**>2 SD**	0.09	0.41	0.59
**>3 SD**	0.02	0.22	0.44
**>5 SD**	0.00	0.04	0.00
**>7 SD**	0.00	0.04	0.00

Variation	To self TVA+VA	To site TVA+VA	To all 27 TVA+VA
**<2 SD**	0.77	0.33	0.22
**>2 SD**	0.23	0.67	0.78
**>3 SD**	0.10	0.52	0.59
**>5 SD**	0.02	0.37	0.33
**>7 SD**	0.01	0.11	0.19

Variation	To self TA_ad	To site TA+adN	To all 27 TA_ad
**<2 SD**	0.78	0.63	0.52
**>2 SD**	0.22	0.37	0.48
**>3 SD**	0.08	0.19	0.33
**>5 SD**	0.01	0.11	0.11
**>7 SD**	0.00	0.07	0.11

Variation	To self HP	To site HP	To all 27 HP
**<2 SD**	0.69	0.41	0.30
**>2 SD**	0.31	0.59	0.70
**>3 SD**	0.10	0.52	0.70
**>5 SD**	0.00	0.37	0.41
**>7 SD**	0.00	0.15	0.15

Variation	To self SSA	To site SSA	To all 27 SSA
**<2 SD**	0.68	0.41	0.22
**>2 SD**	0.32	0.59	0.78
**>3 SD**	0.17	0.56	0.70
**>5 SD**	0.10	0.44	0.48
**>7 SD**	0.05	0.26	0.30

Table S2: Fraction that are outliers by diagnosis and self, (hospital) site and group of 27 pathologists that read >600 colorectal polyp specimens (CRPS). This table is synthesized from Table 1b, Table 2b and Table 2d. The "to self" column is based on 15 pathologists over 7 years; pathologist are compared to their mean call rate. The "to site" comparison is based on 27 pathologists at three hospital sites; each pathologist is compared to the group median diagnostic rate (GMDR) for the hospital. In "to all 27", pathologists are compared to the GMDR of the whole group of 27.

Table S3: Median Call Rates by Hospital Site and Group of 27 Pathologists

Group	HGD	HP	SSA	TVA+VA	TA+ad	HP+SSA	Lump ad	Left CR	Mid CR	Right CR
Site 1	0.020	0.308	0.023	0.076	0.488	0.329	0.553	0.586	0.203	0.192
Site 2	0.009	0.266	0.007	0.043	0.528	0.284	0.579	0.544	0.211	0.236
Site 3	0.026	0.224	0.031	0.068	0.540	0.224	0.604	0.566	0.215	0.207
Group of 27	0.017	0.268	0.019	0.061	0.527	0.282	0.578	0.562	0.209	0.221

Table S3: Median diagnostic call rates by group. "Site 1", "Site 2" and "Site 3" are the three hospital sites. "Group of 27" is the 27 pathologists that interpreted >600 CRPS each. "Lump ad" is the lumping of the "traditional" adenomas (TA+TVA+VA+ad). "Left CR" is the left colon/ rectum. "Mid CR" is the transverse colon and the flexures. "Right CR" is the right colon/cecum.
